# Management of Pyometra Using a Novel Image-Guided Percutaneous Technique: A Case Report

**DOI:** 10.3390/medicina59040689

**Published:** 2023-03-31

**Authors:** Abdulaziz Mohammad Al-Sharydah, Abdulaziz Abdullah AlMulhim, Ibrahim Abdulhakim Al Shikmubarak, Abdulmohsen Ahmed Alhussaini, Raghad Sulaiman Alehaideb, Refaat Salman

**Affiliations:** 1 Department of Diagnostic and Interventional Radiology, King Fahd Hospital of the University, Imam Abdulrahman Bin Faisal University, Khobar P.O. Box: 4398 (31952), Saudi Arabia; 2 Medical Imaging Department, Ministry of Health, Alahsa Health Cluster, Riyadh 11564, Saudi Arabia; 3 King Abdulaziz Hospital, Ministry of National Guard Health Affairs, Riyadh 11564, Saudi Arabia; 4 Vascular Interventional Radiology, Medical Imaging Department, King Abdulaziz Medical City, Ministry of National Guard Health Affairs, Riyadh 11564, Saudi Arabia

**Keywords:** pyometra, collection drainage, balloon dilatation, cervical stenosis/occlusion

## Abstract

Pyometra is a uterine infection that causes pus to accumulate in the uterine cavity. Pyometra primarily affects postmenopausal women. Multiple aetiologies, including cervical stenosis, have been identified. Medical therapy using intravenous antibiotics and surgical evacuation are the conventional treatment options for pyometra. Here, we present a unique case of a novel therapy for pyometra in a geriatric patient; percutaneous alleviation of the causative cervical stenosis was performed using balloon dilatation, along with endometrial drainage of the infected fluid through her vagina, a natural route. This technique has overcome the need for other invasive therapies. The patient’s clinical condition improved significantly after this minimally invasive treatment. Percutaneous balloon dilatation of the cervix for stenosis or occlusion in patients with pyometra facilitates drainage of the infected endometrial fluid. This alternative management technique ensured a satisfactory postoperative course and tolerance in the short-term follow-up. Furthermore, the technique ensured good aesthetic results, with its minimally invasive approach in selected patients, compared to other means of evacuation.

## 1. Introduction

Endometrial fluid collection is associated with benign and malignant conditions and is a concern for malignancy, especially in postmenopausal women [1]. Endometrial fluid collection is generally triggered by either cervical stenosis from an obstructive mass or adhesions related to previous post-inflammatory changes resulting from the aging process [2]. The type of endometrial collection varies in nature; for example, it can be serous (i.e., hydrometra), haemorrhagic (i.e., hematometra), or infected pus (i.e., pyometra) [2].

The incidence of this rare entity ranges from 0.1 to 0.5%, and evidently increases in postmenopausal women by up to 13.6% [3,4,5,6,7].

The clinical diagnosis of this condition is difficult because of its insidious manifestations. A clinical triad of lower abdominal pain, postmenopausal vaginal bleeding, and/or white purulent vaginal discharge has been described [6]. Albeit more than half of those diagnosed with pyometra are asymptomatic [6,8], gynaecological symptoms are generally not evident [9]. Preoperative clinical examinations may reveal signs of generalised rigidity and guarding that is suggestive of peritonitis, thereby reflecting a misleading preoperative diagnosis [6,10].

Sonographic ultrasound plays a pivotal role in the identification and diagnosis of endometrial fluid collection. However, further characterisation can be obtained using cross-sectional imaging techniques (i.e., computed tomography (CT) and magnetic resonance imaging (MRI)), especially for gynaecological diseases that might be associated with oncological aetiologies [2,11].

Endometrial collection has been managed by implementing different techniques, one of which is percutaneous placement of a drainage catheter [1,12,13]. Although antegrade cervical dilatation through the vagina is a known management option for infertile patients caused by cervical stenosis, it can be challenging when accessing the endometrium in an antegrade fashion. Hence, multiple techniques have been used to manage cervical canal stenosis, such as manual dilatation and surgical utilisation of hysteroscopy [14,15,16].

Herein, we report our experience in managing a geriatric patient who was diagnosed with pyometra secondary to cervical stenosis based on cross-sectional imaging and gynaecological examination using a novel minimally invasive technique.

## 2. Case Presentation

The patient was a 72-year-old multiparous woman (all her deliveries were normal, singleton, and through the vagina) with type 2 diabetes mellitus, hypertension, dyslipidaemia, and Alzheimer’s disease. Therefore, she regularly took medications, including metformin (anti-diabetes medication), propranolol (anti-hypertensive medication), atorvastatin (cholesterol-lowering medication), and memantine (glutamate control medication) to control these comorbidities.

The patient presented to the emergency room with complaints of lower abdominopelvic pain, tenderness, and dysuria. The initial diagnosis was a urinary tract infection (UTI) owing to a history of a previously treated UTI; otherwise, she had no history of any other gynaecological condition. She attained menopause at the age of 48 years. She had no history of exposure to radiation, abdominal trauma, or contact with animals.

**Figure 1:**
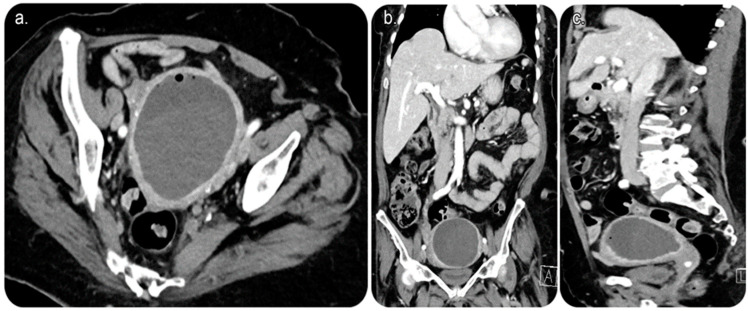
Reformatted computed tomography (CT) images of the abdomen and pelvis with (intravenous) IV contrast. (**a**) Axial cut CT of the pelvis showing an endometrial fluid collection with air focus at the non-dependent part of the endometrium. The endometrial lining shows an evident contrast enhancement. No CT evidence of anterior abdominal scar is seen, proving no prior history of Caesarean birth. (**b**) Coronal and (**c**) sagittal CT of the abdomen and pelvis showing endometrial fluid collection with an air focus at the non-dependent part of the endometrium.

CT of the abdomen and pelvis with intravenous (IV) contrast revealed a sizable endometrial cavity collection with air foci, faint fluid–fluid levelling, and irregular endometrial enhancement. Moreover, an enhanced cervical lesion was identified (Figure 1). Her laboratory results indicated a slight increase in white blood cell count; however, other test results were within the normal range. Pap smear and human papillomavirus tests indicated no evidence of malignancy.

The patient was referred to the Department of Obstetrics and Gynaecology for endovaginal examination. Gynaecological examination confirmed the presence of a cervical lesion obstructing the cervical canal. Another major concern was that no amenable tract was identified that could be used to access the endometrial cavity in an antegrade manner. Considering her age, comorbidities, and general status, and after counselling her family, she was provided palliative management.

The clinical situation warranted a multidisciplinary discussion between obstetricians, gynaecologists, and interventional radiologists to discuss the possible management options. Hence, a mutual decision was made to implement a minimally invasive percutaneous technique, which would be a potentially better option for the patient. This would avoid the problem of a relatively prolonged procedure (e.g., surgical hysterectomy) and increased complication rates, morbidity, and mortality, which is particularly relevant for older patients. Moreover, there were potential hereditary risks associated with general anaesthesia during invasive surgery compared to the minimally invasive percutaneous option.

**Figure 2:**
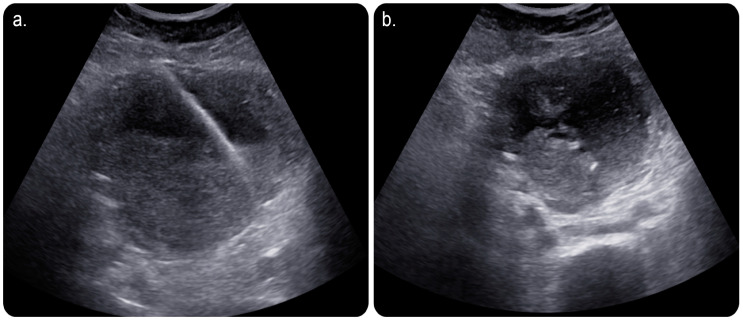
(**a**) Limited grey-scale ultrasound image of the uterus showing a percutaneous echogenic stiff wire inserted through the skin and subcutaneous layers; subsequently, it was secured in the endometrial cavity after an access was obtained using an 18-gauge needle. (**b**) Grey-scale ultrasound image showing a ring-shaped echogenicity at the dependant portion of the collection representing a 10-French-size pigtail catheter looped within the endometrial cavity.

**Figure 3:**
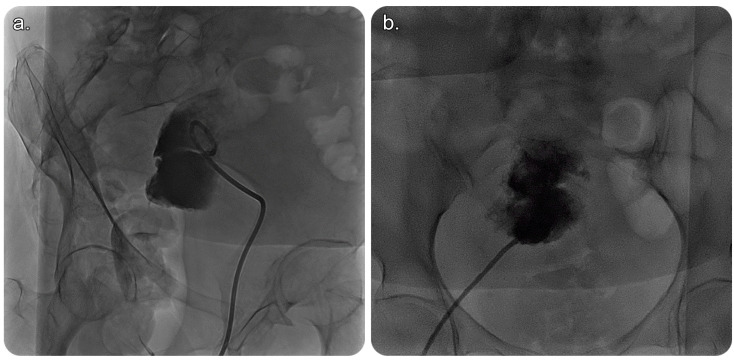
(**a**) Fluoroscopic image of the pelvis in an oblique view showing a 10-French-size pigtail catheter within the endometrial cavity that is filled with contrast material, with no evidence of contrast passage beyond the stenosed/obstructed cervix into the vagina outside the body. (**b**) Fluoroscopic image (frontal view) showing a 10-French-size pigtail catheter within the endometrial cavity that is filled with contrast with a clear cut-off, which indicates obstruction.

In view of this, the patient was referred to an interventional radiology service for a possible image-guided evacuation of the collection. After obtaining informed consent from the patient and her guardian, we performed an ultrasound-guided percutaneous endometrial drainage of the collection by implementing the dual effects of successive percutaneous retrograde cervical dilation using a non-compliant balloon for stretching the causative cervical stenosis under fluoroscopic guidance; this was followed by retrograde drainage catheter insertion under sonographic guidance in one session (Figure 2 and Figure 3).

A preoperative prophylactic antibiotic was prescribed (Piperacillin–tazobactam 4.5 g IV every 8 h) and continued postoperatively for 5 days.

Stepwise, under ultrasound guidance and after local anaesthetic agent (10 mL of 1% lidocaine) administration, a 10-French-size percutaneous drainage catheter (Argon Medical Devices, Plano, TX, USA) was placed inside the uterine collection using the basic Seldinger technique (i.e., 18-gauge needle followed by a stiff wire insert to support the advancement of the drain catheter). A very thick, purulent yellowish, foul-smelling pus was aspirated; hence, the term pyometra is used, and a sample was sent for laboratory analysis. Cytology was negative for malignant cells; however, only inflammatory cells and lactobacilli were identified in the aspirated fluid sample.

Later on, under fluoroscopic guidance and using the same percutaneous retrograde-fashion access, the obstructed cervix was successfully accessed using a 5-French-size angled catheter and wire combination, followed by dilation using a 10 mm diameter by 4 cm length balloon (MUSTANG, Boston Scientific, Marlborough, MA, USA).

Post-balloon dilatation and free contrast passage through the cervix and vagina were observed. In effect, the patient was relieved of any symptoms contributing to cervical stenosis, with excellent aesthetic results and uneventful outcomes.

**Figure 4:**
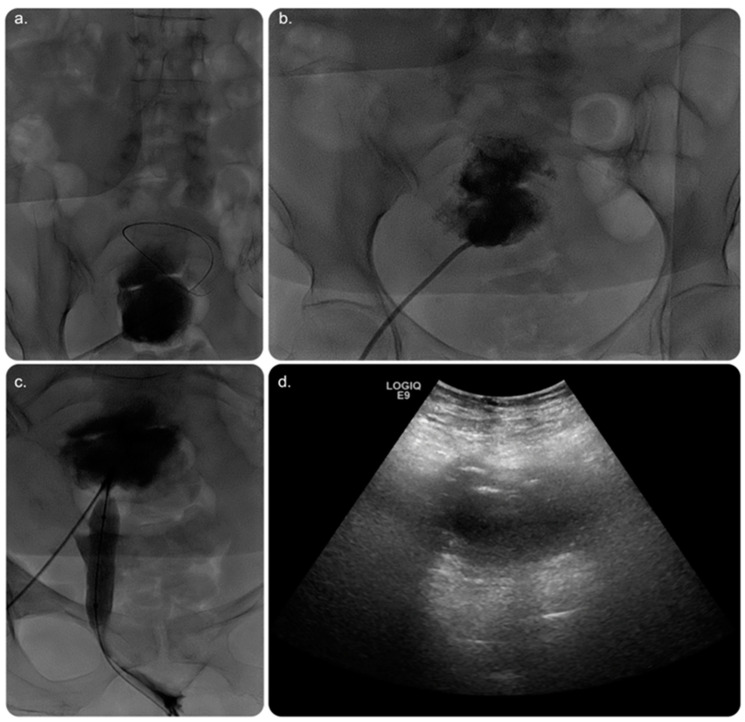
(**a**) Fluoroscopic image of the pelvis showing a wire being introduced through the same access to be directed into the cervix with a trial to negotiate the obstruction. (**b**) Fluoroscopic image showing a catheter being used to direct the wire into the cervix. (**c**) Fluoroscopic image after successfully crossing the cervical stenosis/occlusion with free passage of contrast through the vagina. The wire can be seen projecting through the vagina to outside the body. A stretching balloon was used to dilate the stenosed/obstructed area of the cervix to prevent possible future recurrence of the stenosis. (**d**) Grey-scale ultrasound image performed on day 1 postoperatively, showing significant improvement in the collection with minimal residual amount of the collection.

Day 1 post-procedure, the patient underwent an ultrasound scan, which revealed significant improvement in the endometrial fluid collection. The patient was taken to an Angio Suite, wherein a wire was advanced through the pre-existing drainage catheter that was inserted in a retrograde fashion. Thus, the fluid collection was diverted through the vagina outside the body. The percutaneous drain was removed and replaced after advancing the wire through the cervix until it exited outside the vagina, with another transvaginal catheter inserted over the wire using a similar concept of body flossing technique (Figure 4).

Day 2 postoperatively, an ultrasound scan showed further improvement with no residual fluid. The postoperative leukocyte count was normalised, and all laboratory results were normal. Subsequently, the drain was removed, and the patient was discharged.

After 3 months, the patient was examined in the interventional radiology clinic with no new complaints. Her insidious condition resolved without any complications. Follow-ups in the clinic were scheduled for every six months. The patient’s view on managing her condition was generally satisfactory.

## 3. Discussion

Pyometra is an infection attributed to pus accumulation in the uterine cavity. The literature suggests that over 50% women with unperforated pyometra are asymptomatic [17].

The most predominant symptoms are suprapubic discomfort, fever, chills, postmenopausal bleeding, and purulent vaginal discharge. Pyometra develops when the natural drainage of the uterine cavity is obstructed. Multiple aetiologies have been identified in the literature, including uterine cancer, pelvic inflammatory disease, previous radiation, cervical stenosis, and imperforate hymen.

Among the intriguing findings in this unique case is the positive culture of Lactobacillus acidophilus. Lactobacilli are Gram-positive bacilli that are non-spore-forming, facultative anaerobes that produce lactic acid. Lactobacilli are normally found in the oral flora, gastrointestinal system, and genitourinary tract of females.

Although lactobacilli are generally considered non-pathogenic microbes, some strains are used as probiotics to prevent and treat infections. Furthermore, they are associated with severe clinical infections, including bacteraemia, infective endocarditis, intra-abdominal abscess, postpartum endometritis, and chorioamnionitis [18].

Other causative organisms associated with pyometra have been described in the literature, which commonly include Staphylococci and Streptococci species [8,19]; Escherichia coli, Peptostreptococcus, and Anaerobius bacteroides [5]; and less often, Achromobacter xylosoxidans [19].

The current prospective option for treating endometritis is an image-guided percutaneous pelvic procedure, which often plays an essential role in managing women who endure uterine disorders. This case substantiates the existing literature that promotes image-guided percutaneous drainage of endometrial fluid collection as an effective and safe global procedure [20].

It is performed using ultrasound, fluoroscopy, and/or cross-sectional imaging guidance. Ultrasound guidance is the preferred modality, wherein CT is implemented in cases with deep collections, difficult access, or lesions that cannot be visualised using an ultrasound scan [2] (Figure 1 and Figure 2).

Hence, procedural complications are relatively rare, appearing in only 1–5% of the total performed procedures [1,20]. These few complications include self-limiting haemorrhage, inadvertent bladder injury, temporary abdominal or back pain, temporary tingling in the lower extremities, and infection [1,20].

In contrast, potential complications of pyometra can be catastrophic, including spontaneously perforated pyometra [4,5,6,7,9,10], with subsequent development of bacteraemia [8], generalised peritonitis [5,6], pneumoperitoneum [10], and septic shock [21].

Gynaecological fluid collection can be triggered by a variety of conditions, including appendicitis, diverticulitis, and most commonly, pelvic inflammatory diseases [1]. However, percutaneous drainage has replaced the traditional surgical evacuations, owing to a favourable success rate of 80–85%, with better aesthetic results and low morbidity and mortality rates in contrast to surgical drainage [1].

In our case, percutaneous drainage was incorporated using balloon dilatation of the cervix to treat the underlying cause and naturally drain the fluid collection outside of the body.

Several approaches can facilitate percutaneous drainage of the endometrial fluid collection, including, but not limited to, the transabdominal, posterior transgluteal, transvaginal, and transrectal catheter insertion routes (Figure 3 and Figure 4). The transvaginal route is commonly used, owing to its proximity to the pelvis [1]. In this case, a percutaneous drainage catheter was used to irrigate the endometrial cavity after cervical dilatation to overcome the obstruction. Drain removal depends on multiple factors, such as minimal or no drain, improvement based on imaging, or a decrease in inflammatory markers [1].

Subjectively, the patient was satisfied with the postoperative results without any major complaints. This report has multiple limitations and reflects recent experience with a novel technique to manage pyometra secondary to cervical obstructive lesions; furthermore, our study is a case report and cannot be used to formulate general conclusions.

The insidious course of this diagnostic challenge mandates the implementation of diagnostic strategies to overcome possible oversights from such an entity that could result in a major complication (e.g., spontaneous perforation) [4,5,6,7,9,10] with significant morbidity and mortality.

## 4. Conclusions

Percutaneous balloon dilatation of cervix for stenosis or occlusion in patients with pyometra facilitates drainage of the infected endometrial fluid through a natural route. This alternative management technique ensured a satisfactory postoperative course and tolerance in the short-term follow-up. Moreover, it ensured good aesthetic results with its minimally invasive approach in selected patients, compared to other means of evacuation. Based on the outcomes mentioned earlier, this technique has proven to be a minimally invasive and safe procedure. Therefore, it could be suggested as an alternative to the conventional transvaginal approach, without resorting to general anaesthesia or the need for an operating room. Owing to the insidious course of this rare entity, new diagnostic strategies are required to reduce the potential morbidity and mortality associated with pyometra.

## Data Availability

Data can be obtained from the corresponding author, Refaat Salman, M.D., upon reasonable request.

## References

[1] Li Z., Li L. (2019). The incidence and predictors of gynecologic malignancies among postmenopausal patients with endometrial fluid collection. Arch. Gynecol. Obstet..

[2] Takeuchi M., Matsuzaki K., Uehara H., Yoshida S., Nishitani H., Shimazu H. (2005). Pathologies of the uterine endometrial cavity: Usual and unusual manifestations and pitfalls on magnetic resonance imaging. Eur. Radiol..

[3] Chan L.Y., Lau T.K., Wong S.F., Yuen P.M. (2001). Pyometra. What is its clinical significance?. J. Reprod. Med..

[4] Browne I.L. (2022). Spontaneous perforation of pyometra—Is hysterectomy required in the emergent setting? A case report and literature review. J. Surg. Case Rep..

[5] Yazawa H., Imaizumi K. (2020). Generalized peritonitis secondary to spontaneously perforated pyometra in elderly women: Two cases with different clinical courses and surgical approaches and review of the literature. Fukushima J. Med. Sci..

[6] Malvadkar S.M., Malvadkar M.S., Domkundwar S.V., Mohd S. (2016). Spontaneous rupture of pyometra causing peritonitis in elderly female diagnosed on dynamic transvaginal ultrasound. Case Rep. Radiol..

[7] Sawabe M., Takubo K., Esaki Y., Hatano N., Noro T., Nokubi M. (1995). Occasional Review: Spontaneous Uterine Perforation as a Serious Complication of Pyometra in Elderly Females. Aust. N. Z. J. Obstet. Gynaecol..

[8] Sala A., Restaino S., De Carlo C., Comand M., Frigo A., Rivero S.M., Zanetti E., Driul L. (2021). Postoperative *Streptococcus constellatus* bacteremia in a 75-year-old patient with pyometra: A case report. Am. J. Case Rep..

[9] Huang Y., Tian Q. (2018). Postmenopausal spontaneous rupture of pyometra: A case report. Medicine.

[10] Hashmi M., Ravi G., Khan A. (2023). Spontaneous perforation of uterus presenting with pneumoperitoneum. Eur. J. Mol. Clin. Med..

[11] Goldstein S.R. (1994). Postmenopausal endometrial fluid collections revisited: Look at the doughnut rather than the hole. Obstet. Gynecol..

[12] Álvarez-Sarrado L., Puente-Luján M.J., González-Ballano I., Martínez-Suñer S., Ortega-Marcilla S., Rodríguez-Solanilla B. (2020). Treatment with ultrasound-guided percutaneous drainage of complicated post-cesarean vesicouterine hematomas. Experience in 4 cases. Ginecol. Y Obstet. De México.

[13] Chang S., Sekhon L., Lee J.A., Gounko D., Grunfeld L., Copperman A. (2018). Management of fluid accumulation within the endometrial cavity during a frozen embryo transfer cycle. Fertil. Steril..

[14] Zreik T., Dickey K., Keefe D., Glickman M., Olive D. (1996). Fluoroscopically guided cervical dilatation in patients with infertility. J. Am. Assoc. Gynecol. Laparosc..

[15] Dickey K.W., Zreik T.G., Hsia H.C., Eschelman D.J., Keefe D.L., Olive D.L., Pollak J.S., Rosenblatt M., Glickman M.G. (1996). Transvaginal uterine cervical dilation with fluoroscopic guidance: Preliminary results in patients with infertility. Radiology.

[16] Wood M.A., Kerrigan K.L., Burns M.K., Glenn T.L., Ludwin A., Christianson M.S., Bhagavath B., Lindheim S.R. (2018). Overcoming the challenging cervix: Identification and techniques to access the uterine cavity. Obstet. Gynecol. Surv..

[17] Tay W.M.I., Subramanian M., Chinchure D., Kok S.X.S. (2019). Clinics in diagnostic imaging (199). Singap. Med. J..

[18] Sherid M., Samo S., Sulaiman S., Husein H., Sifuentes H., Sridhar S. (2016). Liver abscess and bacteremia caused by *Lactobacillus*: Role of probiotics? Case report and review of the literature. BMC Gastroenterol..

[19] Mukai S., Isono H., Kondo K., Ihara K., Isono M., Ogawa H. (2022). A Case of Pyometra Caused by Achromobacter xylosoxidans and γ-Streptococcus in an Elderly Frail Woman. Cureus.

[20] Zahedi R., Uppal S., Mendiratta-Lala M., Higgins E.J., Nettles A.N., Maturen K.E. (2016). Percutaneous image-guided pelvic procedures in women with gynecologic cancers: Utilization, complications, and impact on patient management. Abdom. Radiol..

[21] Matsumoto R., Kuramoto S., Muronoi T., Oka K., Shimojyo Y., Kidani A., Hira E., Watanabe H. (2021). Damage control surgery for spontaneous perforation of pyometra with septic shock: A case report. Acute Med. Surg..

